# Thanks

**Published:** 1867-06

**Authors:** 


### *Thanks.*

Sincerest acknowledgements are due and tendered to the public press, for recent flattering notices of the JOURNAL. To the large number of our friends who have also written warm words of encouragement, the editor can only reply, from a full heart, that the JOURNAL is duly grateful.

